# Hypocalcemia in jaundiced neonates receiving phototherapy

**DOI:** 10.12669/pjms.326.10849

**Published:** 2016

**Authors:** Mashal Khan, Kanwal Altaf Malik, Rekha Bai

**Affiliations:** 1 Dr. Mashal Khan, FCPS, MCPS, DCH, Department of Pediatrics and Neonatology, National Institute of Child Health, Karachi, Pakistan; 2 Dr. Kanwal Altaf Malik, MBBS, Department of Pediatrics and Neonatology, National Institute of Child Health, Karachi, Pakistan; 3 Dr. Rekha Bai, MBBS, Department of Pediatrics and Neonatology, National Institute of Child Health, Karachi, Pakistan

**Keywords:** Hypocalcemia, Term neonates, Jaundice, Phototherapy

## Abstract

**Objective:**

To determine the frequency of hypocalcemia in term neonates with jaundice receiving phototherapy.

**Methods:**

This was a cross sectional study conducted at Neonatal intensive care unit, National Institute of Child Health, Karachi from 1st January 2014 to 30th December 2014. A total of 123 term neonates with jaundice of either gender managed by phototherapy were enrolled in the study. Gestational age was assessed through modified Ballard scoring. Duration of phototherapy was recorded. A sample of 3 ml of blood was sent to the laboratory for serum calcium level before initiating phototherapy and after 24 hours of continued phototherapy. All the data were recorded in the preformed proforma. Data was analyzed using SPSS version 19. P value <0.05 was taken as significant.

**Results:**

The mean age of the neonates was 8.35±6.74 days. Mean gestational age at the time of birth was 39.08±1.37 weeks. Mean duration of jaundice was 2.4±1.20 days. Mean duration of phototherapy was 1.74±0.98 days. Serum calcium level before and after 24 hours of initiating phototherapy was 8.73±0.68 mg/dl and 7.47±0.82mg/dl respectively Frequency of hypocalcemia in term jaundiced neonates receiving phototherapy were observed in 22.76% (28/123).

**Conclusions:**

The frequency of hypocalcemia is significant in the jaundiced neonates treated with phototherapy. One needs to be vigilant in dealing neonates in this context while serial monitoring for hypocalcemia and its complications should be considered in institutional policy and research priority.

## INTRODUCTION

Jaundice is a common cause of morbidity encountered in the first week of life. It is an utmost concern for the physician and a source of anxiety for the parents.^1^ High bilirubin level may be toxic to the developing central nervous system and may elicit neurological impairment in newborns.^2^ About 60% of term newborns become visibly jaundiced in the first week of life. In most of the cases, it is benign and no intervention is required.^3^ Approximately 5-10% of them have clinically significant jaundice that signifies the use of phototherapy.^4^

Jaundice is attributable to physiological immaturity of neonates to manage increased bilirubin production. Visible jaundice usually appears between 24-72 hours of age. Basic pathophysiology of jaundice is same in term and preterm neonates, but premature babies are at a higher risk of developing jaundice.^5^ Phototherapy is the most commonly used intervention to treat and prevent severe jaundice. It reduces the risk of exchange transfusion.^6^ It blunts the rise of bilirubin level regardless of the etiology of jaundice.^7^ Though it is considered safe, a few side effects encountered in phototherapy are loose stools, hyperthermia, dehydration due to fluid loss, skin burn, photo retinitis, low platelet count, increased red cell osmotic fragility, bronze baby syndrome, riboflavin deficiency and DNA damage. A lesser known side effect, but a potential complication of phototherapy is hypocalcemia.^8^

The overall prevalence of hypocalcemia in neonates receiving phototherapy was suggested to be 8.7% in full-term newborn.^9^ Another study reflects a little difference between pre and post phototherapy plasma calcium levels (p<0.05).^10^

The relation of hypocalcemia with phototherapy is an important aspect to be considered due to the potential complications of hypocalcemia. This study aimed to cater the burden of hypocalcemia in term neonates treated with phototherapy so as to highlight this aspect, that can further emphasize in modifying local strategies and research priorities.

## METHODS

This cross sectional study was conducted at Neonatal intensive care unit of National Institute of Child Health, Karachi with effect from 1^st^ January 2014 to 30^th^ December 2014. A total of 123 neonates were included in the study. They were term neonates with jaundice requiring phototherapy for at least one day and had a normal calcium level before initiating phototherapy. Informed consent was taken from their parents/guardians. Neonates with jaundice requiring exchange transfusion, birth asphyxia, sepsis, respiratory distress and infants of diabetic mother were excluded from the study. Clearance from institutional ethical review committee was also taken.

Sample size was calculated using software package of WHO. The frequency of hypocalcemia in neonates with jaundice was calculated on the basis of a previous study.^9^ A total of 123 newborn patients were required to achieve 5% level of significance and 5% born on error of estimation. Sampling technique was non probability consecutive sampling. History and examination was performed on every patient. Gestational age was assessed through modified Ballard scoring. Weight and duration of phototherapy was recorded. Hypocalcemia was defined as a total serum calcium level of less than 8 mg/dL in a term neonate.^11^ A sample of 3ml venous blood was sent to the laboratory for serum calcium level before starting conventional phototherapy and after 24 hours of continuous phototherapy. The data was recorded in a predesigned proforma. Data entry and analysis was done using SPSS version 19. Frequency and percentage was computed for categorical variable like sex and hypocalcemic status. Mean and standard deviations was computed for continuous variables like age, gestational age, duration of phototherapy and duration of jaundice. Effect modifiers like age, sex, gestational age, duration of jaundice and duration of phototherapy was controlled through stratification by applying chi squared test and p-value <0.05 was considered significant.

## RESULTS

A total of 123 term neonates included 77 (62.6%) male and 46(37.4%) female. Most of the neonates were < 5 days, with mean age of 8.35±6.74 days. Similarly, mean duration of jaundice was 2.4±1.20 days and the gestational age at the time of birth was 39.08±1.37 weeks (Table-I).

**Table-I:** Descriptive statistics of the age, gestational age and duration of jaundice.

Descriptive statistics		Age (Days)	Gestational Age (Weeks)	Duration of Jaundice (Days)
Mean		8.35	39.08	2.41
Std. Deviation		6.74	1.37	1.20
95% Confidence Interval of Mean	Lower Bound	7.15s	38.84	2.18
	Upper Bound	9.55	39.33	2.61
Median		6	29	2
Interquartile Range		9	2	2

Mean duration of phototherapy was 1.74±0.98 days and serum calcium level before and after phototherapy was 8.73±0.68 mg/ dl and 7.47±0.82mg/dl respectively (Table-II). Frequency of hypocalcaemia in term neonates with jaundice receiving phototherapy was found to be 22.76% (28/123). Stratification analysis was performed and observed that hypocalcaemia was not significant among the different age groups (p=0.86), gender (p=0.117), gestational age (p=0.65), duration of jaundice (p=0.77) and duration of phototherapy (p=0.56) (Table-III).

**Table-II:** Descriptive statistics of serum calcium and duration of phototherapy n=123.

Descriptive statistics		Duration of Phototherapy (Days)	Serum Calcium Level (mg/dl)	
			Pre phototherapy	Post phototherapy
Mean		1.74	8.73	7.47
95% Confidence Interval of Mean	Lower Bound	1.56	8.61	7.86
	Upper Bound	1.92	8.85	8.15
Median		1	8.5	8.10
Std. Deviation		0.98	0.68	0.82
Minimum		1	8	5.1
Maximum		5	11.4	9.85
Interquartile Range		1	1.1	0.70

**Table-III:** Frequency of hypocalcemia with respect to age, gender, gestational age, duration of phototherapy and duration of jaundice.

	Hypocalcaemia		Total

	Yes	No	
*Age Groups (Days)*			
≤ 5 days	13 (22%)	46(78%)	59
5.1 to 10 days	7(22.6%)	24(77.4%)	31
10.1 to 15 days	4(33.3%)	8(66.67%)	12
15.1 to 20 days	3(23.1%)	10(76.9%)	13
> 20 days	1(12.5%)	7(87.5%)	8
*Gender*			
Male	14(18.2%)	63(81.8%)	77
Female	14(30.4%)	32(69.6%)	46
*Gestational Age (Weeks)*			
37+1 to 40	24 (23.5%)	78(76.5%)	102
> 40	4(19%)	17(81%)	21
*Duration of Phototherapy*			
1 day	21(21.6%)	76(78.4%)	97
2 to 5 days	7(26.9%)	19(73.1%)	26
*Duration of Jaundice*			
1 to 3 days	22(22.2%)	77(77.8%)	99
> 3 days	6(25%)	18(75%)	24

Chi square P value is 0.01at 95% confidence interval.

## DISCUSSION

Neonatal jaundice is a frequent cause of morbidity in newborns worldwide and the most frequent cause of hospitalization and readmission in the initial week of life.^12^ Recent global surveys curtails that every year, roughly 1.1 million babies would develop severe neonatal jaundice and the vast majority reside in sub-Saharan Africa and South Asia.^13^

Phototherapy is an appropriate and safe measure to reduce jaundice in a newborn. Romagnoli et al. was the first to suggest the association of hypocalcemia in a newborn following phototherapy.^14^

The regulation of calcium homeostasis in the newborn period is of considerable interest. At birth the plasma calcium level in cord blood exceeds that in maternal blood. During the early days of life, the plasma calcium level progressively decreases in normal infants, so by the 2^nd^ or 3rd day of life, the level is lower than that found in older infants. In full term infants the plasma calcium level returns to normal by 10 days of life.^15^ The mechanism of hypocalcemic effect of phototherapy was reported by inhibition of pineal gland via transcranial illumination, resulting in decline of melatonin secretion that further inhibits the effect of cortisol on bone calcium. Cortisol has direct hypo calcemic effect and increases bone uptake of calcium and induces hypocalcemia.^16^ Hunter et al. also hypothesized this mechanism.^17^ Eghbalian F further established this phenomenon in his study, exhibiting a direct relation between duration of phototherapy and the development of hypocalcemia.^10^

In our study the mean age of the neonates and gestational age at the time of birth was 8.35±6.74 days and 39.08±1.37 weeks. There were 77 (62.6%) male and 46 (37.4%) female. This matches the study by Kramifer H et al. that included the mean age of 5.69±2.6 days.^18^

Our study shows 22.76% (28/123) of term neonates exhibited phototherapy induced hypocalcemia. In Iran, a study reflects 7% of term neonates with phototherapy induced hypocalcemia.^19^ Ehsanipoor et al. and Karamifar et al. reported the incidence of hypocalcemia to be 15% and 8.7% respectively.^20^,^21^ Jain BK et al. after 48 hours of continuous phototherapy observed hypocalcemia in 30% of term neonates.^22^ In contrast much higher frequency was observed by Yadavs, about 66.6% of neonates developed hypocalcemia after phototherapy.^9^ Also Medhat found hypocalcemia in 75% of term neonates after phototherapy.^23^ A Latest study by Bahbah et al. done in 2014 at Egypt studied only 50 term neonates who received phototherapy for jaundice and 25 neonates were taken as controls with physiological jaundice needing no phototherapy. After 48 hours of phototherapy hypocalcemia was found in 26%.^24^

The effectiveness of phototherapy in the management of neonatal jaundice is well known. The efforts made round the globe recognize it as a potential complication with variable results some reflecting more severe hypocalcemia than others, considering the duration of phototherapy exposure, the severity of hyperbilirubinemia when phototherapy was initiated and also the term neonate versus preterm neonates. All these factors add to the severity of hypocalcemia induced by phototherapy. Our study explains a significant effect of phototherapy on the calcium balance in a term neonate with jaundice, receiving phototherapy. Further studies are needed to elaborate this aspect further, to suggest if calcium supplementation were to given in neonates who receive phototherapy for neonatal jaundice.

## CONCLUSIONS

Our study concludes that a significant number of neonates i.e. 22.6% developed hypocalcemia after phototherapy. The range of hypocalcemia varied from very low levels to just borderline decrease in total calcium. This in turn can have clinical impact and adds to the morbidity. Hence this study is helping us many folds, to raise our concern towards another impact of phototherapy while alleviating jaundice on other hand. This adds to our institutional policies and research priorities.
